# NLRP3 Inflammasome as a Potentially New Therapeutic Target of Mesenchymal Stem Cells and Their Exosomes in the Treatment of Inflammatory Eye Diseases

**DOI:** 10.3390/cells12182327

**Published:** 2023-09-21

**Authors:** Carl Randall Harrell, Valentin Djonov, Ana Antonijevic, Vladislav Volarevic

**Affiliations:** 1 Regenerative Processing Plant, LLC, 34176 US Highway 19 N, Palm Harbor, FL 34684, USA; dr.harrell@regenerativeplant.org; 2 Institute of Anatomy, University of Bern, Baltzerstrasse 2, 3012 Bern, Switzerland; valentin.djonov@ana.unibe.ch; 3 Center for Harm Reduction of Biological and Chemical Hazards, Faculty of Medical Sciences, University of Kragujevac, 69 Svetozar Markovic Street, 34000 Kragujevac, Serbia; ana.ant89@gmail.com; 4 Department of Genetics and Department of Microbiology and Immunology, Faculty of Medical Sciences, University of Kragujevac, 69 Svetozar Markovic Street, 34000 Kragujevac, Serbia

**Keywords:** NLRP3 inflammasome, mesenchymal stem cell, exosomes, therapy, inflammatory eye diseases

## Abstract

Due to their potent immunoregulatory and angio-modulatory properties, mesenchymal stem cells (MSCs) and their exosomes (MSC-Exos) have emerged as potential game-changers in regenerative ophthalmology, particularly for the personalized treatment of inflammatory diseases. MSCs suppress detrimental immune responses in the eyes and alleviate ongoing inflammation in ocular tissues by modulating the phenotype and function of all immune cells that play pathogenic roles in the development and progression of inflammatory eye diseases. MSC-Exos, due to their nano-sized dimension and lipid envelope, easily bypass all barriers in the eyes and deliver MSC-sourced bioactive compounds directly to target cells. Although MSCs and their exosomes offer a novel approach to treating immune cell-driven eye diseases, further research is needed to optimize their therapeutic efficacy. A significant number of experimental studies is currently focused on the delineation of intracellular targets, which crucially contribute to the immunosuppressive and anti-inflammatory effects of MSCs and MSC-Exos. The activation of NLRP3 inflammasome induces programmed cell death of epithelial cells, induces the generation of inflammatory phenotypes in eye-infiltrated immune cells, and enhances the expression of adhesion molecules on ECs facilitating the recruitment of circulating leukocytes in injured and inflamed eyes. In this review article, we summarize current knowledge about signaling pathways that are responsible for NLRP3 inflammasome-driven intraocular inflammation and we emphasize molecular mechanisms that regulate MSC-based modulation of NLRP3-driven signaling in eye-infiltrated immune cells, providing evidence that NLRP3 inflammasome should be considered a potentially new therapeutic target for MSCs and MSC-Exo-based treatment of inflammatory eye diseases.

## 1. Introduction

Inflammatory eye diseases encompass a diverse group of conditions characterized by inflammation in various structures of the eye [1]. These conditions can affect different parts of the eye, including the conjunctiva (conjunctivitis), cornea (keratitis), uvea (anterior/intermediate/posterior uveitis, pan-uveitis), retina (retinitis, retinal vasculitis), and optic nerve (optic neuritis) [1]. Inflammatory eye diseases may be caused by microbial pathogens, trauma, chemical irritants, and allergens, or could develop as a consequence of autoimmune reactions, due to immune cell-driven tissue damage [1]. Dry eyes represent the main symptom of Sjögren’s syndrome; bulging eyes and double vision are frequently observed in patients suffering from Graves’ disease; and redness, pain, sensitivity to light, and blurred vision are the main ocular manifestations of rheumatoid arthritis and systemic lupus erythematosus (SLE) [1].

Therapy for inflammatory and autoimmune eye diseases focuses on managing the underlying condition and reducing inflammation in the eyes [2]. Treatment plans vary depending on the specific condition and severity of the disease [2]. Eye drops or ointments containing corticosteroids, non-steroidal anti-inflammatory drugs (NSAIDs), or immunosuppressive agents are usually applied directly to the eye to reduce mild inflammation and alleviate conjunctivitis and keratitis-related symptoms [3]. In more severe cases, oral medications such as corticosteroids, immunosuppressants, or biologics may be used to suppress detrimental immune responses and to prevent the progression of ongoing eye inflammation [3]. For certain conditions, such as severe uveitis, injections of corticosteroids or immunosuppressive agents may be administered directly into the eye. In some cases, surgical procedures such as vitrectomy or cataract surgery may be necessary to restore vision and to treat complications caused by inflammatory or autoimmune eye diseases [3]. Although therapeutic strategies for the treatment of inflammatory eye diseases have evolved significantly, there are still some issues that limit the therapeutic efficacy of these approaches [2,3]. Prolonged use of corticosteroids may increase the risk of cataracts or glaucoma while long-term use of immunosuppressive drugs may inhibit the immune system throughout the body, increasing the risk of infections. Since inflammatory and autoimmune eye diseases manifest differently in each individual, identifying the most suitable treatment approach often requires a personalized approach [2,3]. Finally, achieving complete remission or long-term control of the underlying autoimmune or inflammatory disease can be challenging. Some patients may require ongoing treatment and monitoring to manage their symptoms and prevent relapses. Accordingly, a large number of experimental and clinical studies are continuously conducted in order to resolve limits and to improve therapeutic options for the treatment of inflammatory eye diseases [2,3].

Due to their potent immunoregulatory and angio-modulatory properties, mesenchymal stem cells (MSCs) have emerged as potential game-changers in regenerative ophthalmology, particularly in the field of the treatment of inflammatory diseases [4]. MSC-based therapy targets the underlying cause of inflammation rather than just managing disease-related symptoms [4]. MSCs have been shown to exert their therapeutic effects via several mechanisms. Firstly, MSCs suppress detrimental immune responses in the eyes and alleviate ongoing inflammation in ocular tissues by modulating the phenotype and function of all immune cells that play pathogenic roles in the development and progression of inflammatory and autoimmune eye diseases (monocytes/macrophages, dendritic cells (DCs), natural killer (NK), natural killer T (NKT) cells, and T and B lymphocytes) [4]. 

Additionally, MSCs possess potent anti-angiogenic properties, which can be beneficial in the treatment of neovascularization-associated inflammatory eye diseases, such as diabetic retinopathy and age-related macular degeneration [5]. By inhibiting the formation of abnormal blood vessels in the retina, MSCs can help preserve vision and prevent further damage to the ocular tissues [5]. 

Clinical studies investigating the therapeutic potential of MSCs in inflammatory eye diseases have shown promising results. A study conducted on patients with refractory uveitis demonstrated that the intravenous infusion of MSCs led to a significant reduction in ocular inflammation and improvement in visual acuity [6]. Similarly, in dry eye disease (DED), stem cell-based therapy was shown to restore lacrimal gland dysfunction, improve tear production, reduce ocular surface damage, and alleviate DED-related symptoms [7].

Although MSCs offer a novel approach to treating immune cell-driven eye diseases, further research is needed to optimize their therapeutic efficacy [6]. A significant number of experimental studies is focused on the delineation of signaling pathways and intracellular targets in immune cells, which crucially contribute to the immunosuppressive and anti-inflammatory effects of MSCs [8]. NLRP3 inflammasome, composed of Nod-like receptor family pyrin domain-containing protein 3 (NLRP3), apoptosis-associated speck-like protein containing a caspase activation and recruitment domain (CARD) (ASC), and pro-caspase-1 is dysregulated in almost all inflammatory eye disease [9]. Accordingly, a large number of experimental findings suggested that this multi-protein complex could be considered an important intracellular target for MSC-based therapy of inflammatory eye disorders [9]. In order to emphasize the role of NLRP3 inflammasome in MSC-dependent attenuation of ocular inflammation, in this review article, we summarize current knowledge about signaling pathways that are responsible for the pathogenic role of NLRP3 inflammasome in the development and progression of inflammatory eye diseases, and we emphasized molecular mechanisms, which are involved in MSC-based modulation of NLRP3-driven signaling in eye-infiltrated immune cells.

## 2. Methodology

An extensive literature review was carried out in July 2023 across several databases (MEDLINE, EMBASE, and Google Scholar) from 1990 to the present. Keywords used in the selection were “mesenchymal stem cells”, “exosomes”, “NLRP3 inflammasome”, “signaling pathway”, “eye inflammation”, “immunosuppression”, “immunoregulation”, “autoimmunity”, and “cell-based therapy”. All journals were considered, and an initial search retrieved 217 articles. The abstracts of all these articles were subsequently reviewed by two of the authors (CRH and VV) independently to check their relevance to the subject of this manuscript. Discussions were held to settle any disputes. If a compromise could not be reached, a third researcher (VD), who was an authority on the subject matter of the study, arbitrated the dispute. The Grading of Recommendations, Assessment, and Evaluation (GRADE) method created by the Cochrane Collaboration was used to evaluate the strength of the evidence for the studies that were ultimately included. Eligible studies had to delineate molecular and cellular mechanisms responsible for the MSC-based modulation of NLRP3-driven eye inflammation, and their findings were analyzed and discussed in this review. 

## 3. Activation of NLRP3 Inflammasome in Eye-Infiltrated Immune Cells as an Initial Step in the Development of Inflammatory Eye Disease

The activation of the NLRP3 inflammasome in eye-infiltrated immune cells leads to the enhanced production of pro-inflammatory cytokines (interleukin-1β (IL-1β) and IL-18), which elicit inflammatory processes in ocular structures [10].

**Figure 1:**
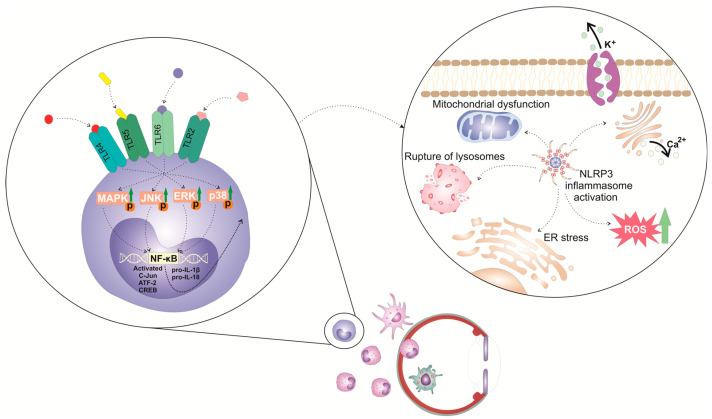
Molecular mechanisms and signaling pathways involved in the activation of NLRP3 inflammasome in eye-infiltrated immune cells. The activation of the NLRP3 inflammasome in immune cells is a tightly regulated, multi-step process. It requires 2 steps: priming and activation. The priming step of NLRP3 inflammasome activation is elicited by the activation of pattern recognition receptors (PRRs), including Toll-like receptors (TLR). These membrane-bound receptors are expressed on immune cells that recognize pathogen-associated molecular patterns (PAMPs) of microbial antigens or damage-associated molecular patterns (DAMPs) released from injured cells. Signals generated from activated TLR-2, TLR-4, TLR-5, and TLR-6 initiate phosphorylation and consequent activation of extracellular signal-regulated kinase (ERK), c-Jun N-terminal kinase (JNK), and p38 mitogen-activated protein kinase (MAPK), which, in turn, phosphorylate c-Jun, ATF-2, and CREB transcription factors that bind to the promoter regions of NLRP3 and pro-IL-1β, leading to their transcriptional upregulation.

The activation of the NLRP3 inflammasome in immune cells is a tightly regulated, multi-step process (Figure 1). It requires two steps: priming and activation. Since some immune cells do not naturally express pro-IL-1 and have inadequate amounts of NLRP3 for inflammasome activation, one of the main functions of the priming step is to stimulate the transcriptional production of NLRP3, pro-IL-1β, and pro-IL-18. This initial step involves the activation of transcriptional factors (the nuclear factor kappa B (NF-κB), c-Jun, ATF-2, and CREB) that, upon activation, translocate to the nucleus to induce the enhanced transcription of pro-inflammatory genes, resulting in the increased synthesis of NLRP3 and pro-IL-1β. The priming step of NLRP3 inflammasome activation is elicited by the activation of pattern recognition receptors (PRRs), including Toll-like receptors (TLR). These membrane-bound receptors are expressed on immune cells where recognized pathogen-associated molecular patterns (PAMPs) of microbial antigens or damage-associated molecular patterns (DAMPs) are released from injured cells [10,11]. Signals generated from activated PRRs, particularly TLR-2, TLR-4, TLR-5, and TLR-6, initiate phosphorylation and the consequent activation of extracellular signal-regulated kinase (ERK), c-Jun N-terminal kinase (JNK), and p38 mitogen-activated protein kinase (MAPK), which, in turn, phosphorylate c-Jun, ATF-2, and CREB transcription factors that bind to the promoter regions of NLRP3 and pro-IL-1β, leading to their transcriptional upregulation [10,11]. Additionally, upon binding to TLRs, TLR ligands elicit the MyD88-driven activation of IL-1R-associated kinase 1 (IRAK-1), which, in a TNFR-associated factor 6 (TRAF6)-dependent manner, induces the activation of transforming growth factor-beta-activated kinase 1 (TAK-1), a key protein kinase responsible for the optimal activation of NF-κB. Activated TAK-1 phosphorylates and activates the IκB kinase (IKK) complex, which consists of IKKα, IKKβ, and IKKγ (also known as NEMO). The activated IKK complex phosphorylates the Inhibitor of κB (IκB), which, within cytosol, binds to NF-κB and prevents its nuclear translocation. The phosphorylation of IκB leads to its degradation via the ubiquitin–proteasome pathway. This degradation releases NF-κB, allowing it to translocate into the nucleus. Once in the nucleus, NF-κB binds to specific DNA sequences called κB sites, promoting the transcription of NF-κB-dependent genes, such as NLRP3, pro-IL-1β, and pro-IL-18, which are necessary for inflammasome activation.

In addition to the priming step, a second step, often referred to as the “activation signal” or the “danger signal” is necessary for the full and optimal activation of the NLRP3 inflammasome. This second step is driven by NLRP3 agonists, which activate NLRP3 to cause inflammasome assembly and mature IL-1 production. It can be triggered by a variety of stimuli, including microbial components, extracellular matrix breakdown products, and environmental antigens [10,11,12]. The exact mechanisms by which these stimuli activate the NLRP3 inflammasome are still not fully understood, but proposed mechanisms include alterations in potassium (K^+^) efflux, lysosomal rupture, mitochondrial dysfunction, and enhanced reactive oxygen species (ROS) production [10,11]. Increased intracellular levels of ATP, massive accumulation of uric acid crystals, microbial antigens, and environmental irritants can induce potassium (K^+^) efflux from the immune cell, leading to decreased intracellular potassium levels. NLRP3 contains a potassium (K^+^)-sensing domain that undergoes conformational changes in response to altered potassium levels [10,11]. Accordingly, a decrease in intracellular potassium levels leads to conformational changes in the NLRP3 protein, allowing its oligomerization and consequent association with adaptor ASC protein made via homotypic interactions between the pyrin domains of these two molecules. NLRP3:ASC interaction leads to the formation of a large protein complex called the inflammasome [10,11]. NIMA-related kinase 7 (NEK7), an important component of the NLRP3 inflammasome complex, stabilizes the NLRP3 protein and promotes its interaction with ASC protein. Once the NLRP3 inflammasome complex is formed, the CARD domain of ASC protein interacts with the CARD domain of pro-caspase-1, facilitating its recruitment to the inflammasome complex [12]. Afterward, pro-caspase-1 undergoes self-cleavage and activation, resulting in the formation of active caspase-1. Active caspase-1 then cleaves pro-IL-1β and pro-IL-18 into their mature forms, IL-1β and IL-18. These two cytokines are important inflammatory mediators that crucially contribute to the generation and propagation of all inflammatory eye diseases [10,11]. 

In addition to cytokine release, caspase-1 activation also leads to the cleavage of gasdermin D (GSDMD), an effector protein involved in the execution of pyroptosis, a highly inflammatory form of programmed cell death, which has an important pathogenic role in the development and progression of ocular inflammation [13]. NLRP3 and caspase-1-dependent cleavage of GSDMD results in the formation of two distinct fragments of GSDMD protein: the N-terminal (GSDMD-N) and the C-terminal domains (GSDMD-C). Once released from the C-terminal domain, GSDMD-N oligomerizes and inserts into the lipid bilayer of the plasma membrane, leading to the formation of large pores called gasdermin pores [13]. Gasdermin pores disrupt the integrity of the plasma membrane, causing the efflux of intracellular ions, cytoplasmic proteins, and pro-inflammatory cytokines (IL-1β and IL-18) into the extracellular space. IL-1β and IL-18 propagate and aggravate inflammation initially elicited by the activation of NLRP3 inflammasome and importantly contribute to the creation of a “positive inflammatory loop” in inflamed eyes that finally results in enhanced activation and the increased accumulation of inflammatory immune cells in inflamed eyes [13].

## 4. Molecular Mechanisms Responsible for NLRP3/IL-1β/IL-18-Dependent Generation of Detrimental Immune Response in Inflamed Eyes

NLRP3/IL-1β/IL-18-dependent recruitment of immune cells is a complex process involving the activation of endothelial cells (ECs), the release of chemotactic factors, the modulation of adhesion molecules, and changes in vascular dynamics (Table 1) [9]. NLRP3-generated IL-1β and IL-18 induce increased expression of adhesion molecules (intercellular adhesion molecule-1 (ICAM-1), vascular cell adhesion molecule-1 (VCAM-1), E and P-selectins) on the membrane of ECs [9]. Additionally, the activation of NLRP3 inflammasome in immune cells enhances the affinity and avidity of integrin–ligand interactions and facilitates firm adhesion of immune cells to ECs. Precisely, the NLRP3/IL-1β/IL-18 axis upregulates the expression of integrins (lymphocyte function-associated antigen-1 (LFA-1) and very late antigen-4 (VLA-4)) on immune cells, which interact with their ligands on ECs, further promoting the adhesion and subsequent transmigration of immune cells into the inflamed tissues of affected eyes [9]. 

The NLRP3/IL-1β/IL-18 signaling pathway promotes vasodilation and induces increased vascular permeability in inflamed eyes [14]. Vasodilation leads to the widening of blood vessels, allowing more immune cells to flow closer to the site of inflammation [14]. Increased vascular permeability allows immune cells to extravasate from the bloodstream into the tissue spaces, facilitating their recruitment and accumulation in the inflamed eyes [14]. 

Additionally, the activation of NLRP3 inflammasome induces massive production of inflammatory cytokines (tumor necrosis factor alpha (TNF-α), IL-6, and IL-8), chemokines (CCL2, CCL3, CCL5, CXCL8, and CXCL10), and prostaglandins in eye-infiltrated monocytes and lymphocytes [10,11]. These inflammatory mediators act synergistically with IL-1β and IL-18 to promote the recruitment of inflammatory immune cells from the bloodstream to the site of eye inflammation [10,11].

Also, the NLRP3/IL-1β/IL-18 pathway importantly contributes to tissue remodeling in inflamed eyes [18]. This axis stimulates the production of matrix metalloproteinases (MMPs) in immune cells, ECs, and fibroblasts. MMP-2, MMP-3, and MMP-9 degrade the extracellular matrix components, including collagen and proteoglycans, leading to tissue damage and breakdown of structural integrity [18]. The NLRP3-dependent increase in intraocular concentration of IL-1β and IL-18 disrupts the integrity of the blood-aqueous barrier and blood-retinal barrier, which normally prevents the free passage of cells and molecules between the blood and the eye [18]. In this way, NLRP3/IL-1β/IL-18 signaling facilitates the migration of immune cells across the barriers. This leads to increased vascular permeability and the leakage of fluid, proteins, and cells into the ocular tissues, exacerbating the ongoing inflammation. Also, the NLRP3/IL-1β/IL-18-dependent disruption of blood-aqueous and blood-retinal barriers may result in complications such as cystoid macular edema, retinal detachment, and glaucoma [18].

Finally, the NLRP3/IL-1β/IL-18 axis is essential for shaping the adaptive immune response and for promoting effective T and B cell-driven immune defense against eye-invading pathogens [19]. Accordingly, the dysregulation of the NLRP3/IL-1β/IL-18 pathway can lead to an aberrant adaptive immune response and contribute to the development of autoimmune diseases and chronic inflammation in the eyes [19].

**Figure 2:**
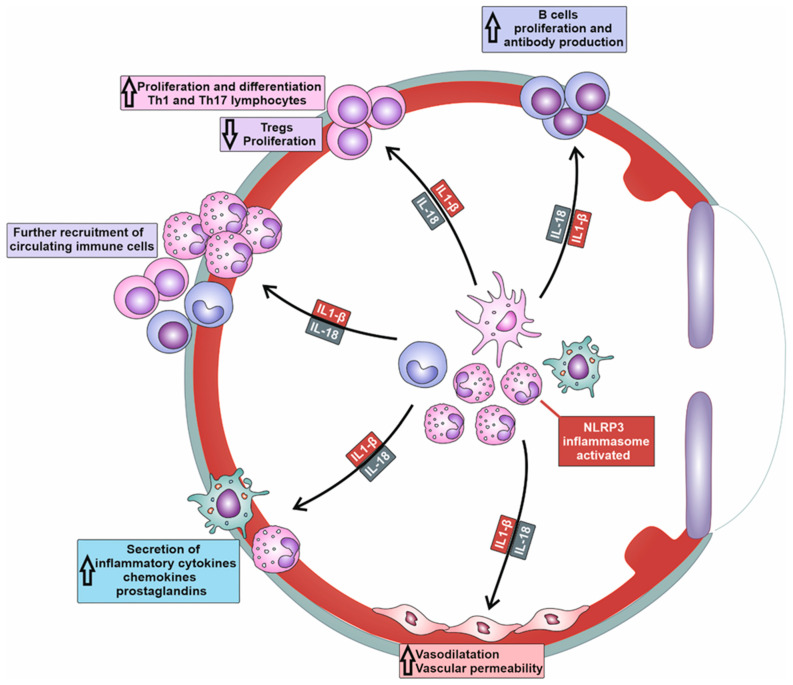
Molecular mechanisms responsible for NLRP3-dependent generation of detrimental immune response in inflamed eyes. Activation of NLRP3 inflammasome results in increased production of IL-1β and IL-18 in tissue-infiltrated macrophages and neutrophils. IL-1β and IL-18 induce massive recruitment of circulating leukocytes in inflamed tissues, increased expansion of inflammatory Th1 and Th17 lymphocytes, enhanced production of inflammatory cytokines and chemokines in neutrophils and macrophages, attenuated proliferation of immunosuppressive T regulatory cells (Tregs), and increased antibody production in plasma cells.

When the NLRP3 inflammasome is activated, IL-1beta and IL-18 are released, activating naive T cells and promoting their proliferation and clonal expansion (Figure 2). The NLRP3/IL-1β/IL-18 axis can synergize with other co-stimulatory molecules, such as CD28, to amplify T cell activation signals [10,15]. NLRP3/IL-1β/IL-18 acts as a co-stimulatory signaling pathway that enhances the activation of T cell receptors (TCRs) on T cells, leading to increased production of cytokines and cytotoxic molecules. The NLRP3/IL-1β/IL-18-dependent activation of T cells contributes to the expansion of antigen-specific T cell responses and the generation of an effective adaptive immune response [10,11,15].

Importantly, the NLRP3/IL-1β/IL-18 axis can influence the differentiation of T cells, particularly CD4+ T helper cells [15]. IL-1β and IL-18 promote the differentiation of naïve CD4+ T cells in effector, pro-inflammatory T helper 1 (Th1) cells and Th17 cells, which are mainly responsible for the T cell-driven injury of corneas, conjunctiva, lacrimal, and meibomian glands in patients suffering from severe keratitis, uveitis, conjunctivitis, and DED [15]. IL-1β and IL-18 act synergistically with other inflammatory (pro-Th1 and pro-Th17) cytokines such as IL-12 and IL-23 to induce the expression of lineage-specific transcription factors (e.g., T-bet for Th1 cells and RORγt for Th17 cells) and the production of effector cytokines (e.g., IFN-γ for Th1 cells and IL-17A for Th17 cells) [10,11,15]. Th1 and Th17 lymphocytes are inflammatory cells that play a crucially important pathogenic role in the development of DED. Th1 cells produce IFN-γ, which increases the cytotoxic properties of NK cells and enhances the production of nitric oxide (NO), ROS, and inflammatory cytokines in macrophages and DCs, improving their antigen-presenting properties. Th17 cell-derived IL-17 generates inflammatory phenotypes in eye-infiltrated neutrophils and promotes neutrophil-driven tissue injury in inflamed eyes, lacrimal, and meibomian glands of DED patients [10,11,15].

Additionally, the NLRP3/IL-1β/IL-18 axis may inhibit the generation and suppressive function of Tregs, which play a crucial role in immune tolerance within the eyes [16]. NLRP3/IL-1β/IL-18 signaling can suppress the expression and activity of the transcription factor forkhead box P3, (Foxp3), which is critical for Treg development and function [16]. By attenuating the generation of immunosuppressive Tregs, the NLRP3/IL-1β/IL-18 axis promotes the progression of detrimental immune response in inflamed eyes, crucially contributing to the aggravation of ongoing inflammation [16].

In addition to cellular immunity, NLRP3/IL-1β/IL-18 signaling controls the activation of B cells and regulates antibody production, importantly contributing to the development of humoral immune response against microbial pathogens [17]. The NLRP3/IL-1β/IL-18 axis initiates co-stimulatory signals for B cell activation, enhancing B cell receptor (BCR) downstream pathways and increasing the secretory profile of plasma cells [17]. The NLRP3/IL-1β/IL-18 axis can induce the increased expression of activation-induced cytidine deaminase (AID), which is involved in class-switch recombination and somatic hypermutation, leading to the production of different antibody isotypes and high-affinity antibodies [16,17]. Accordingly, the NLRP3/IL-1β/IL-18 signaling pathway plays an important role in the development and progression of antibody-dependent autoimmune eye diseases, including Graves’ ophthalmopathy, Myasthenia gravis, Ocular Cicatricial Pemphigoid, and SLE-related DED [15,17].

In line with all these findings, the targeting of the NLRP3/IL-1β/IL-18 axis in immune cells has emerged as a potentially new therapeutic approach that could further enhance the efficacy of immunoregulatory agents, including MSCs, in the therapy of inflammatory and autoimmune eye disorders [9].

## 5. MSC-Sourced Factors That Are Responsible for MSC-Based Suppression of NLRP3 Inflammasome in Immune Cells

The immunomodulatory properties of MSCs make them an attractive therapeutic tool for the alleviation of detrimental immune responses in inflamed eyes [4,8]. Via the secretion of immunoregulatory factors, the induction of indoleamine 2,3-dioxygenase (IDO) signaling, and by the immunomodulatory activity of microRNAs (miRNAs), MSCs exert their suppressive effects on the NLRP3 inflammasome in immune cells, leading to the reduced synthesis of pro-inflammatory cytokines IL-1β and IL-18 in inflamed tissues, resulting in the alleviation of ongoing inflammation (Table 2) [4,8].

**Figure 3:**
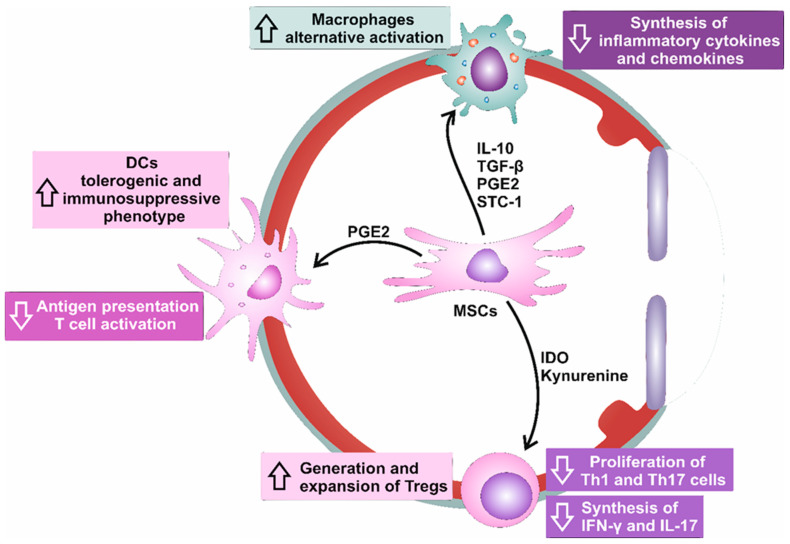
The beneficial effects of MSCs and MSC-derived exosomes in the attenuation of NLRP3-driven eye injury and inflammation. MSCs secrete a wide array of immunosuppressive cytokines and immunoregulatory factors, such as IL-10, transforming growth factor-β (TGF-β), prostaglandin E2 (PGE2), and stanniocalcin (STC)-1, which efficiently suppress NLRP3 inflammasome activation and inhibited production of IL-1β and IL-18 in eye-infiltrated macrophages. MSC-derived PGE2 was found mainly responsible for the MSC-dependent generation of tolerogenic phenotype in DCs, which was followed by attenuated activation of naïve T cells. MSC-sourced IDO and Kynurenine were considered mainly responsible for the MSC-dependent regulation of T/NK/NKT cells’ phenotype and function.

MSCs secrete a wide array of immunosuppressive cytokines and immunoregulatory factors, such as IL-10, transforming growth factor-β (TGF-β), and prostaglandin E2 (PGE2), which efficiently suppress NLRP3 inflammasome activation and the inhibited production of IL-1β and IL-18 in eye-infiltrated macrophages, which results in attenuated corneal injury and inflammation (Figure 3) [4,8]. Similarly, MSC-sourced PGE2 reduces the synthesis of IL-1β and IL-18 by inhibiting the TGF-activated kinase 1 (TAK1)/NF-kβ-dependent activation of NLRP3 inflammasome in macrophages [20]. As a result of NLRP3 inflammasome suppression, macrophages exposed to MSC-derived PGE2, obtain immunosuppressive (M2) phenotypes (characterized by increased expression of *Arg1*, *Mgl1*, *Mgl2*, and *Ym1* M2-macrophage associated genes) and, instead of inflammatory IL-1β and IL-18, mainly produce anti-inflammatory IL-10 [20]. 

Oh and colleagues suggested that MSC-sourced stanniocalcin (STC)-1 was mainly responsible for the MSC-dependent modulation of macrophages’ phenotype and function. STC-1 is an immunoregulatory protein that downregulates the expression of all NLRP3 components (NLRP3, ASC, and pro-caspase-1) and inhibits activation of NF-kβ and MAPK-driven signaling pathways in macrophages [22]. NLRP3-activated macrophages stimulate MSCs to increase the expression and secretion of stanniocalcin STC-1, while, in turn, MSCs in an STC-1-dependent manner, prevent activation of NLRP3 inflammasome by suppressing the generation of mitochondrial ROS in activated macrophages [22]. 

In a similar manner as it was observed in macrophages, MSCs, via the inhibition of NLRP3 inflammasome, induce the generation of tolerogenic and immunosuppressive phenotypes in DCs, as well [21]. MSC-derived PGE2 was found mainly responsible for the MSC-dependent suppression of NLRP3 inflammasome in DCs, which was followed by the downregulated expression of co-stimulatory molecules (CD80 and CD86) and by the reduced production of inflammatory cytokines (TNF-α, IFN-γ, IL-1β, and IL-18) [21]. Importantly, by reducing the activation of NLRP3 inflammasome in DCs, MSCs significantly attenuate their antigen-presenting properties and alleviate their capacity for the induction of T cell-driven inflammation [21]. 

Accordingly, the MSC-based suppression of NLRP3/IL-1β/IL-18-dependent activation of eye-infiltrated NK, NKT cells, Th1, and Th17 lymphocytes crucially contributes to the attenuation of T and NK/NKT cell-driven inflammatory eye diseases, including DED, herpetic stromal keratitis, uveitis, and Graves’ disease [4,8,21]. MSC-sourced IDO, an enzyme that catalyzes tryptophan metabolism, was considered mainly responsible for MSC-dependent regulation of T/NK/NKT cells’ phenotype and function [23]. MSC-sourced IDO depletes tryptophan in the inflamed microenvironment [23]. The reduced availability of tryptophan affects the metabolic balance and alters the intracellular amino acid sensing mechanisms, impacting the activation of the NLRP3 inflammasome in immune cells [23]. To be precise, the IDO-dependent reduction of L-tryptophan inhibits the mechanistic target of the rapamycin (mTOR) signaling pathway, which reduces the activation of NLRP3 inflammasome. Additionally, metabolites generated from tryptophan metabolism, such as kynurenine and quinolinic acid, suppress ROS production and inhibit the activation of the NLRP3 inflammasome [23,24]. Also, kynurenine and its downstream metabolite 3-hydroxyanthranilic acid activate the aryl hydrocarbon receptor (AhR), which, in turn, inhibits NLRP3 inflammasome activation and attenuates the subsequent production of IL-1β and IL-18 in eye-infiltrated immune cells [23,24]. Finally, MSC-sourced IDO enhances the generation and expansion of immunosuppressive Tregs since kynurenine promotes the expression of Treg lineage-defining transcription factor FoxP3 in naïve CD4+ T cells. Upon activation of TCRs, intracellular signaling pathways driven by protein kinase B and mTOR destabilize the immunoregulatory phenotype of resting Tregs and cause their reprogramming into a pro-inflammatory helper-like phenotype (‘ex-Tregs’), characterized by increased production of Th1 and Th17-related inflammatory cytokines, IFN-γ and IL-17 [23,24]. By inducing low levels of tryptophan in the local microenvironment, MSC-derived IDO and kynurenine activate stress-response-induced general control nonderepressible 2 (GCN2) kinase, which suppresses protein kinase B/mTOR signaling in Tregs and prevents the trans-differentiation of Tregs in inflammatory Th1 and Th17 cells [24,25]. Additionally, since GCN2 kinase downregulates the expression of TCR zeta chain in CD8+ cytotoxic T cells (CTLs), by increasing the activity of GCN2 kinase in CTLs, MSC-sourced IDO and kynurenine attenuate cytotoxic properties of CTLs and suppress CTL-dependent damage of epithelial cells in inflamed conjunctiva and lacrimal glands of experimental animals suffering from DED [24,25].

## 6. Beneficial Effects of MSCs and MSC-Derived Exosomes in the Attenuation of NLRP3-Driven Eye Injury and Inflammation

Several experimental studies demonstrated that the beneficial effects of MSCs in the treatment of inflammatory eye diseases relied on their capacity to suppress NLRP3 inflammasome-driven injury of epithelial cells [27,28,29]. 

By analyzing molecular mechanisms responsible for MSC-based protection of sodium iodate (NaIO3)-injured retinal pigment epithelial cells (RPECs), Mao and colleagues concluded that MSCs prevent apoptosis of injured RPECs by suppressing NF-κB-mediated NLRP3 inflammasome activation [27]. MSCs improved the viability of NaIO3-treated RPECs by preventing oxidative stress-mediated mitochondrial disruption. MSCs preserved the integrity of the mitochondrial membrane, reduced the activity of pro-apoptotic Bax protein, and increased the activity of anti-apoptotic Bcl-2 protein in NaIO3-exposed RPECs [27]. Significantly downregulated expressions of NF-κB, attenuated activation of NLRP3 inflammasome, diminished activity of caspase-1/3/8, and reduced synthesis of inflammatory IL-1β were observed in NaIO3-injured RPECs that were cultured with MSCs, indicating that MSC-dependent suppression of NF-kB-mediated NLRP3 inflammasome was mainly responsible for RPECs survivability. Importantly, Mao and colleagues showed that MSCs prevented NLRP3-driven apoptosis of RPECs in a contact-independent manner, suggesting that bioactive factors contained in MSC-sourced secretome were responsible for their beneficial effects [27]. 

MSC-derived secretome is enriched with MSC-derived exosomes (MSC-Exos), extracellular vesicles that contain the majority of MSC-sourced bioactive compounds. MSC-Exos, due to its lipid envelope and nano-sized dimension, easily bypass all barriers in the body, including blood-aqueous and blood-retinal barriers and therefore represent novel therapeutic agents in MSC-based therapy of inflammatory eye diseases [5]. MSC-Exos carry a cargo of MSC-sourced immunoregulatory proteins (IL-10, TGF-β) that can enter the cytoplasm of corneal epithelial cells and eye-infiltrated leukocytes, affecting their phenotype and function [5,24,25]. MSC-sourced IL-10 induces the generation of immunosuppressive phenotype in eye-infiltrated macrophages and DCs, while MSC-derived TGF-β induces G1 cell cycle arrest in activated T lymphocytes, NK, and NKT cells via the inhibition of the Jak/STAT pathway, preventing the development and progression of immune cell-driven eye injury and inflammation [24,25].

In addition to immunosuppressive cytokines, MSC-Exos also contain a large number of MSC-sourced microRNAs (MSC-miRNAs), which modulate protein synthesis in target eye-infiltrated immune cells via the post-transcriptional regulation of target mRNA [25]. Zhang and colleagues recently demonstrated that MSC-Exo-containing miR-146a represents one of the major regulators of NLRP3 inflammasome activation and pro-inflammatory cytokine secretion in macrophages [30]. MSC-derived miR-146a is able to efficiently suppress NLRP3 inflammasome activation in inflammatory immune cells, crucially contributing to the alleviation of ongoing inflammation [30,31]. MSC-sourced miR-146a directly targets and binds to the 3’ untranslated regions (UTRs) of NLRP3 and ASC proteins, two key components of the NLRP3 inflammasome complex. The binding of miR-146a to the 3’ UTRs of NLRP3 and ASC leads to the degradation of their mRNA, resulting in reduced expression levels of these proteins [31]. MSC-derived miR-146a also targets and suppresses the expression of downstream signaling molecules involved in the activation of the NLRP3 inflammasome [26]. It directly inhibits the expression of interleukin-1 receptor-associated kinase 1 (IRAK)-1 and TNF receptor-associated factor 6 (TRAF6), which are key mediators in the TLR and interleukin-1 receptor (IL-1R)-driven signaling pathways. By targeting these molecules, miR-146a dampens the inflammatory signaling cascade that leads to NLRP3 inflammasome activation. Also, MSC-sourced miR-146a can indirectly inhibit NLRP3 inflammasome activation by targeting and suppressing the expression of IRAK-2, which is involved in the NF-κB signaling pathway. By downregulating NF-κB signaling, MSC-derived miR-146a indirectly suppresses the transcriptional activation of NLRP3 and prevents the synthesis of NF-κB-driven pro-inflammatory cytokines IL-1β and IL-18 [26]. It should be noted that miR-146a is also part of a negative feedback loop that regulates the NLRP3 inflammasome activation in ECs and immune cells [32]. MiR-146a expression is induced upon the stimulation of immune cells with pro-inflammatory signals, such as lipopolysaccharide (LPS) or cytokines (IFN-γ and TNF-α). Increased miR-146a levels then act to suppress the expression of NLRP3, ASC, IRAK1, and TRAF6, thereby limiting the NLRP3 inflammasome activation and subsequent production of pro-inflammatory cytokines in eye-infiltrated immune cells. In this way, MSC-derived miR-146a importantly contributes to the suppression of chronic inflammatory response in the eyes [32].

The therapeutic potential of MSC-Exos to avoid biological barriers in the eye and to deliver immunosuppressive factors directly in eye-infiltrated immune cells was used for the development of MSC-Exo-containing eye drops that showed beneficial effects in the attenuation of inflammatory eye diseases [28]. Yu and colleagues used a murine model of DED to demonstrate that MSC-Exo-containing eye drops efficiently suppressed NLRP3-driven apoptosis of corneal epithelial cells, inhibited NLRP3-driven activation of eye-infiltrated immune cells, and attenuated desiccation-induced ocular surface damage [28]. Significantly reduced corneal epithelial defects, increased tear production, decreased goblet cell loss, and the downregulated production of inflammatory cytokines were observed in the eyes of MSC-Exo-treated mice. The topical administration of MSC-Exo-containing eye drops protected corneal epithelial cells against hyperosmotic stress (HS)-induced cell apoptosis [28]. The downregulated expression of NLRP3-related genes (*NLRP3, ASC, caspase-1*) and reduced concentration of inflammatory IL-1β and IL-18 were observed in corneal and conjunctival epithelia of MSC-Exo-treated mice suffering from desiccating stress, suggesting that MSC-Exos prevented ocular surface damage and attenuated DED progression by suppressing HS-induced formation of NLRP3 inflammasome [28]. By inhibiting activation of NRLP3 inflammasome, MSC-Exos prevented the NLRP3/caspase-1-dependent apoptosis of corneal epithelial cells and inhibited the NLRP3/IL-1β/IL-18-driven generation of detrimental immune response and inflammation in the eyes of experimental animals [28]. 

Similar findings were reported by Wang and colleagues who demonstrated beneficial effects of MSC-Exos in the alleviation of benzalkonium chloride (BAC)-induced mouse model of DED [29]. The topical administration of MSC-Exos suppressed the activation of NLRP3 inflammasome and inhibited NLRP3-driven apoptosis of corneal epithelial cells. The inhibition of NLRP3 inflammasome led to the attenuated activity of caspase-1, which prevented caspase-1-dependent programmed cell death of corneal epithelial cells. By suppressing NLRP3 inflammasome, MSC-Exos inhibited the production of inflammatory cytokines (IL-1β and IL-18) in eye-infiltrated leukocytes [29]. Furthermore, suppression of the NLRP3/IL-1β/IL-18 axis induced the generation of tolerogenic and anti-inflammatory phenotypes in immune cells, which resulted in the downregulated production of inflammatory cytokines and upregulated synthesis of immunosuppressive IL-10 [29]. Accordingly, significantly decreased concentrations of IL-1β, IL-18, TNF-α, IL-6, IL-1α, and IFN-γ and an increased concentration of IL-10 were observed in the eyes of BAC-treated mice that received MSC-Exos, suggesting that MSCs prevented the progression of BAC-induced corneal injury and inhibited progression of DED by suppressing NLRP3-driven eye inflammation [29].

## 7. The Use of MSCs and Their Exosomes in Clinical Settings

Due to their huge therapeutic potential, MSCs and MSC-Exos have been used in several clinical trials to attenuate ongoing NLRP3-driven eye inflammation.

A double-blinded randomized clinical trial that investigated the therapeutic efficacy of adipose tissue-derived MSCs (AT-MSCs) in the treatment of severe aqueous deficient dry eye disease (ADDE) due to Sjögren’s Syndrome (SS) was recently completed in Denmark (NCT04615455). According to the study protocol, 40 patients were randomly divided into experimental or control groups to receive either AT-MSCs or a placebo (vehicle, Crystore CS10). Allogeneic AT-MSCs (22 million cells/mL), previously obtained from healthy donors, were transconjunctivally injected into the lacrimal gland of one eye. Differences in ocular surface disease index (OSDI), non-invasive keratography tear break-up time (NIKBUT), tear meniscus height (TMH), tear secretion, tear osmolarity, and staining of the ocular surface were compared between the experimental and control groups of patients during the 4 months of follow-up. Donor-specific MHC-antibodies were determined in the blood of AT-MSC-treated patients in order to detect induction of detrimental, allogeneic immune response in the recipients of MHC-mismatched AT-MSCs. This clinical study was completed in January 2023 and the obtained results are not published yet.

The safety and efficacy of intravenous and sub-tenon delivery of allogeneic umbilical cord-derived mesenchymal stem cells (UC-MSCs) for the treatment of eye diseases will be examined in a clinical trial conducted in Argentina, Antigua, and Barbuda (NCT05147701). According to the study protocol, patients will receive a total dose of 100 million UC-MSCs. Possible adverse events or complications of UC-MSCs will be monitored during the four-year follow-up. The study is currently recruiting patients, and the first results are expected after January 2026.

The effects of Umbilical Mesenchymal Stem Cell (uMSC)-derived Exosomes (uMSC-Exos) on the alleviation of DED-related symptoms in patients with chronic Graft Versus Host Diseases (cGVHD) have been evaluated in a clinical study, which is currently being conducted at Sun Yat-sen University, China (NCT04213248). According to the study protocol, 27 cGVHD patients with DED will initially receive artificial tears for 2 weeks to obtain the normalized baseline. Afterward, patients will topically receive uMSC-Exos (10ug/drop) four times a day for 14 days. The changes in (i) ocular surface disease index (OSDI) score (ii) tear secretion amount, (iii) tear break time, (iv) ocular surface staining, (v) best corrected visual acuity (BCVA), (vi) conjunctiva redness score, and (vii) tear meniscus height will be regularly determined for 12 weeks. This study currently recruits patients, and the estimated study completion date is December 2023.

## 8. Conclusions and Future Perspectives

NLRP3 inflammasome, which plays a crucially important pathogenic role in the development and progression of inflammatory eye diseases should be considered a potentially new therapeutic target for the MSC-based treatment of inflammatory eye diseases. MSCs suppress the activation of NLRP3 inflammasome in a paracrine manner, via the activity of MSC-sourced IL-10, TGF-β, PGE2, IDO, and miRNA-146, preventing activation of detrimental immune response in the eyes. Due to their capacity to avoid biological barriers, MSC-Exos are able to deliver MSC-sourced bioactive factors directly to injured epithelial cells and eye-infiltrated leukocytes, suppressing NLRP3-dependent apoptosis, pyroptosis, and IL-1β/IL-18-driven inflammation. Although results of animal studies demonstrated the beneficial effects of MSC-Exo-containing eye drops in the suppression of NLRP3/IL-1β/IL-18-caused eye inflammatory eye diseases, new clinical studies should be conducted to confirm these findings before MSC-Exos can be offered as new therapeutic agents in the treatment of NLRP3-driven inflammatory eye diseases.

## Figures and Tables

**Table 1:** The role of inflammatory cytokines and chemokines in the development and progression of NLRP3 inflammasome-driven eye inflammation.

Cytokine/Chemokine	Cell Source	Mechanisms of Action	Biological Effects	Ref. No.
IL-1β, IL-18, TNF-α, CCL2, CCL3, CCL5, CXCL8, CXCL10	monocytes/macrophages DCs neutrophils	increased expression of adhesion molecules on the membrane of ECs within inflamed eyes	increased recruitment of circulating leukocytes in inflamed eyes	[9]
IL-1β, IL-6, IL-8, IL-18, PGE2	monocytes/macrophages DCs neutrophils	Increased vascular permeability	increased extravasation and accumulation of inflammatory immune cells in inflamed eyes	[13]
IL-1β and IL-18	monocytes/macrophages	increased production of MMP-2, MMP-3 and MMP-9	tissue damage and disruption of blood-aqueous and blood-retinal barriers	[14]
IL-1β, IL-18, IL-12, IL-23	DCs	activation of naïve T cells and their differentiation in effector, inflammatory Th1 and Th17 cells	generation of Th1 and Th17 cell-driven eye inflammation	[10,11,15]
IL-1β and IL-18	DCs	suppressed expression and activity of the transcription factor FoxP3 in T cells	attenuated expansion of Tregs	[16]
IL-1β, IL-6, IL-18	monocytes/macrophages DCs neutrophils	increased AID expression	increased production of high-affinity antibodies	[17]

Abbreviations: interleukin (IL), tumor necrosis factor alpha (TNF-α), C–C motif chemokine ligand (CCL), C–X-C motif chemokine ligand (CXC), dendritic cells (DCs), endothelial cells (ECs), matrix metalloproteinase (MMP), T regulatory cells (Tregs), forkhead box P3, (Foxp3), activation-induced cytidine deaminase (AID).

**Table 2:** Regulation of NLRP3 inflammasome by MSCs and MSC-derived exosomes in eye injury and inflammation.

MSC/MSC-Exo-Sourced Factor	Target Cells	Effect(s)	Ref. No.
IL-10, TGF-β, PGE2	macrophages	generation of immunosuppressive phenotype; inhibited production of IL-1β and IL-18; attenuated corneal injury and inflammation	[4,8]
PGE2	DCs	generation of tolerogenic phenotype; increased production of IL-10	[20,21]
STC-1	macrophages	attenuated generation of ROS	[22]
IDO	activated T lymphocytes, NK, and NKT cells	suppressed proliferation	[23]
TGF-β	activated T lymphocytes NK and NKT cells	G1 cell cycle arrest	[24]
IDO/Kynurenine	Tregs	enhanced expansion	[24,25]
IDO/Kynurenine	CTLs	suppress CTL-dependent damage of epithelial cells in inflamed conjunctiva and lacrimal glands	[24,25]
miR-146a	macrophages, DCs, neutrophils	downregulation of NF-κB signaling; attenuated production of IL-1β and IL-18	[26]

Abbreviations: prostaglandin E2 (PGE2), transforming growth factor beta (TGF-β), dendritic cells (DCs), interleukin (IL), stanniocalcin (STC)-1, indoleamine-2,3-dioxygenase (IDO), reactive oxygen species (ROS), natural killer cells (NK), natural killer T cells (NKT), T regulatory cells (Tregs), cytotoxic T lymphocytes (CTLs), micro-RNA (miR), Nuclear factor kappa B (NF-κB).
